# Frequency-Domain Characteristics Response to Passive Exercise in Patients With Coronary Artery Disease

**DOI:** 10.3389/fcvm.2021.760320

**Published:** 2021-12-14

**Authors:** Xiaodong Zhang, Yahui Zhang

**Affiliations:** ^1^Department of Physical Education, Nanjing University of Finance and Economics, Nanjing, China; ^2^Department of Cardiology, The Eighth Affiliated Hospital of Sun Yat-sen University, Shenzhen, China

**Keywords:** passive exercise, ultrasonic spectrum image, frequency-domain characteristics, arterial hemodynamics, coronary artery disease

## Abstract

**Purpose:** The enhanced external counterpulsation (EECP), a kind of passive exercise, is a novel non-invasive therapy used to improve peripheral perfusion in patients with coronary artery disease (CAD). However, whether frequency-domain characteristics of peripheral hemodynamics may benefit from passive exercise needs to be verified.

**Methods:** We recruited 21 patients with CAD and 21 healthy controls in this study. Ultrasonic blood flow velocity spectrum in left carotid (LC) and right carotid (RC) common arteries, and right brachial (RB) and right femoral (RF) arteries was monitored using an ultrasonic Doppler. Frequency-domain characteristics before, during, and after passive exercise were extracted from ultrasonic spectrum images. The first and second peak amplitudes/frequencies (y1, y2, x1, x2) and power spectral energy ratio (PSER) in the 0–2.05 Hz/0.87 Hz (p5, p6) were calculated by fast Fourier transform and power spectrum density analysis.

**Results:** For the amplitude and frequency characteristics of the spectrum, y1 in the LC of patients with CAD was significantly decreased during exercise (*p* = 0.036), whereas, y2 was significantly decreased immediately after passive exercise (*p* = 0.038). Besides those, y1 only in the RC and RB of controls was significantly decreased during exercise. Immediately after exercise, y2 in the LC of control was significantly lower than at the baseline (*p* = 0.014). For the energy ratio characteristics of the spectrum, there was an opposite response in the two groups that p6 was significantly reduced and elevated in the LC of controls and in the RB of patients with CAD during exercise (both *p* < 0.05).

**Conclusions:** Passive exercise reduces amplitude and frequency characteristics of carotid arteries, while there was an opposite response of energy ratio characteristics in the LC and RB arteries to passive exercise between CAD patients and controls. Additionally, energy ratio characteristics of spectrum in the brachial artery were markedly elevated in CAD patients during passive exercise. Moreover, passive exercise only reduces amplitude characteristics of LC artery in the control group.

## Introduction

Previous studies have found that Doppler power frequency spectrum characteristics analysis can improve the early diagnosis of carotid artery disease and detect early small flow disturbances in carotid arteries (1). Additionally, a study has also reported that spectral descriptors of blood velocity waveforms can be a better indicator of preclinical microvascular abnormalities (2). Moreover, some studies have found that low-frequency characteristics included the information necessary to describe the kinetics of blood flow (3) and sympathetic activity (4). Furthermore, during exercise, the total power of pulse and the 1st and 2nd pulse harmonics were significantly increased, whereas, after exercise, the power of the 2nd harmonics was also increased in Tai Chi Chuan practitioners (5).

The enhanced external counterpulsation (EECP) is regarded as a kind of passive exercise to alleviate symptoms of angina and reduce myocardial ischemia (6, 7). Studies have reported that the EECP not only significantly increased the coronary blood flow velocity in patients with coronary artery disease (CAD) (8, 9), but also markedly elevated peripheral blood flow (10–12). Moreover, our previous study investigated that EECP can improve the hemodynamic variables in the carotid and peripheral arteries (13). However, little is known about the responses of frequency-domain characteristics of conduit artery hemodynamics, such as carotid and peripheral arteries, which contain important information to describe the kinetics of cardiovascular function (3).

To comprehensively investigate the response of conduit artery hemodynamics to passive exercise, we extracted and calculated the frequency-domain characteristics of ultrasonic spectrum image, such as peak amplitude, corresponding frequency, and energy ratio. This study aimed to investigate the acute responses of passive exercise on frequency-domain characteristics of ultrasonic spectrum image in the carotid, brachial, and femoral arteries in patients with CAD, and to compare the responses to those of controls to further explore the mechanisms underlying these responses.

## Materials and Methods

### Participants

We enrolled 21 in-patients at the Eighth Affiliated Hospital of Sun Yat-sen University with CAD, diagnosed by angiographically proven stenosis ≥50% in at least one major coronary artery. Twenty-one healthy people were enrolled as controls. Exclusion criteria were contraindications for EECP, such as III hypertension (systolic BP ≥ 180 mmHg and/or diastolic BP ≥ 100 mmHg), carotid dissection, aortic aneurysm, severe lower extremity venous thrombosis, and severe systemic disease and malignancy. Measurements described below were performed on the CAD patients and controls before, during, and after a 45-min session of EECP treatment. The study was approved by the local medical ethics committee of the Eighth Affiliated Hospital of Sun Yat-sen University, and written informed consent was obtained from all participants.

### Design

All subjects are received with a single, 45-min session of EECP. Subjects lay supine on the EECP treatment bed with their legs and buttocks wrapped in cuffs. They were sequentially inflated from the lower thigh to the upper thigh and buttocks at the start of the diastole phase, followed by a fast, simultaneous deflation of all cuffs just before the onset of systole. Passive exercise-EECP was conducted using an Oxygen saturation monitoring enhanced external counterpulsation instrument (Pushikang P-ECP/TM, Chongqing, China). The counterpulsation pressure was set as 0.028–0.033 MPa for both the groups.

### Measurement of Peripheral Vascular and Blood Flow Characteristics

The color Doppler ultrasound (GE logic E) examination was conducted 10 min before the beginning of the passive exercise, at 15–25 min after its start, and 30 s−1 min immediately after the end of the session. The measurement time for each section was about 2 min. Measurement order was right carotid (RC), left carotid (LC), right brachial (RB), and femoral artery (RF). The right and left common carotid arteries (CCAs) were scanned 15 mm proximal to the internal–external carotid bifurcation. RB and RF measurement sites were fixed ~50 mm above the antecubital fossa and 20 mm below the inguinal ligament, respectively. The RF artery was not measured during passive exercise because it was wrapped in the cuffs with the legs.

### Data Analysis

#### Image Processing to Obtain the Velocity Spectrum

**Figure 1:**
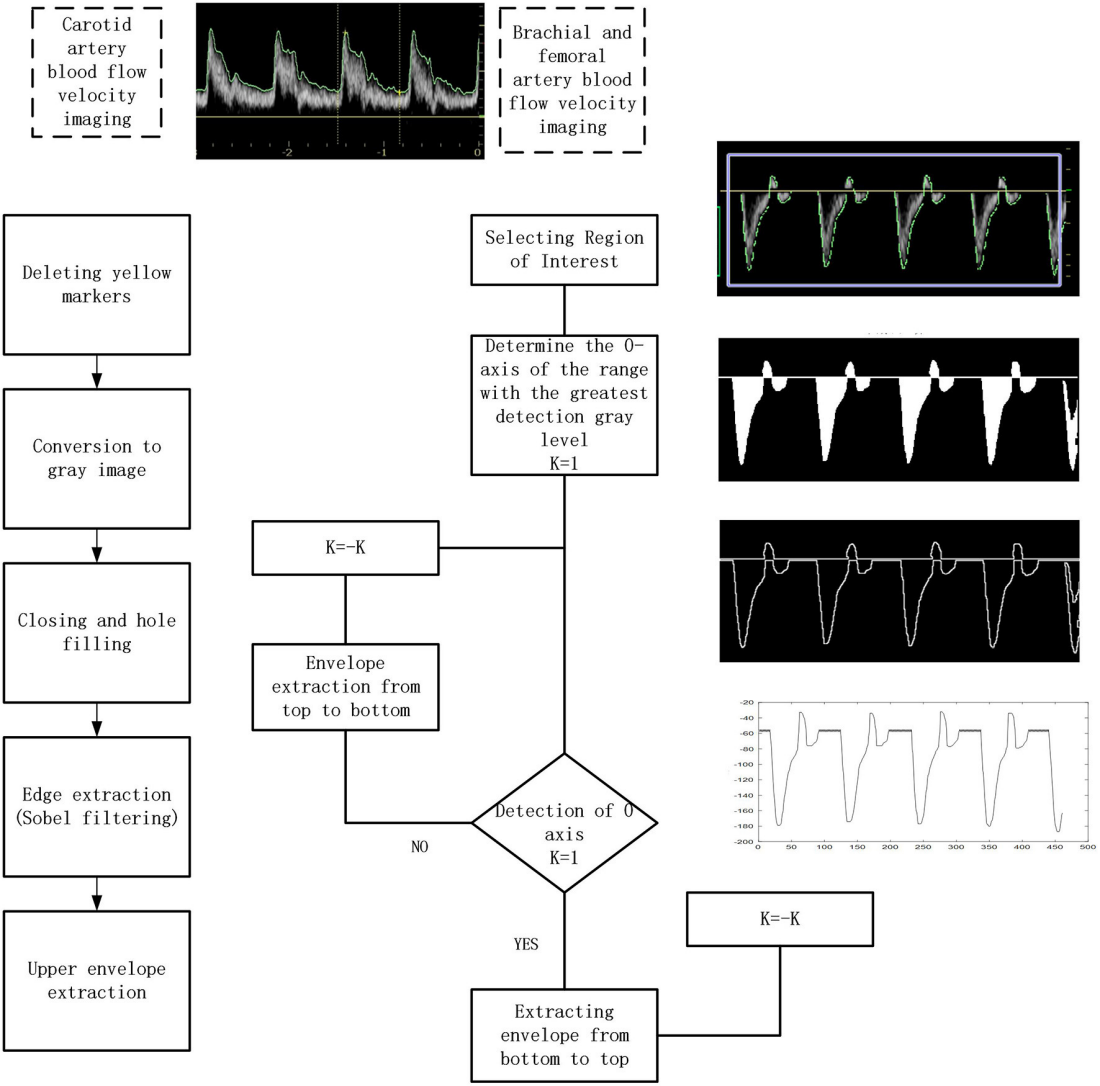
Flowchart of the image processing.

A flowchart of the procedure is shown in Figure 1. Various algorithms were applied to the blood flow velocity traces from the carotid, brachial, and femoral arteries. The main steps included removal of the yellow markers produced by the scanner's integral software, converting the image to gray-scale, closing and hole filling, and Sobel filtering to extract edges followed by envelope extraction. Additionally, the velocity spectrum of brachial and femoral arteries was inverted about the zero-flow axis to orientate the traces.

#### Feature Extraction

##### Amplitude and Frequency Characteristics

The first and second peak amplitude (y1, y2), corresponding with first and second peak frequencies (x1, x2), were extracted from the spectrum curve. The frequency-domain features of the blood flow signals from the brachial, femoral, and carotid arteries were analyzed by the Fast Fourier Transform (FFT). The spectrum was obtained from the following expression (14):


(1) $$\begin{array}{l} X\left(k\right)=\overset{N-1}{\underset{n=0}{\sum }}x\left(n\right)W_{N}^{kn}\;k=0,1,...,N-1,\;W_{N}=e^{-j\frac{2\pi }{N}}, \end{array}$$

where *x(n)* is the blood flow velocity signal (*n* = 0, 1, …, *N*-1), and *N* is sampling point.

##### Energy Ratio Characteristics

**Figure 2:**
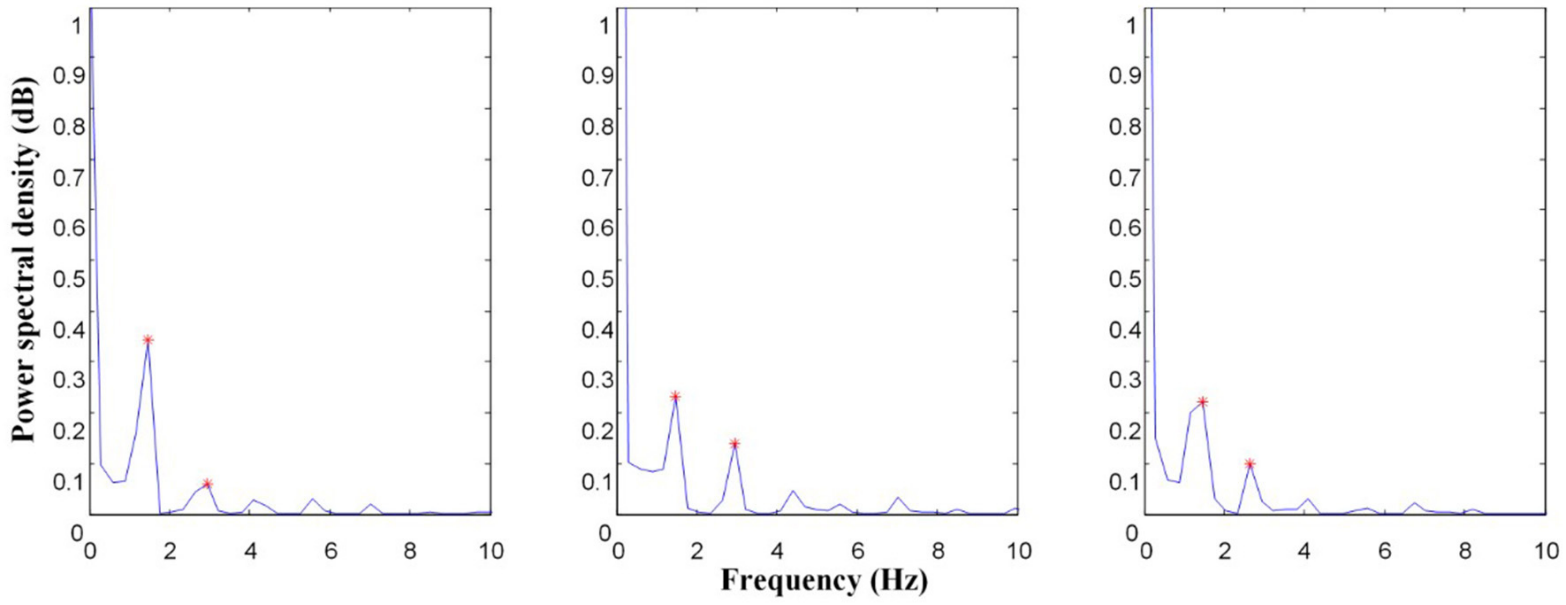
Power spectrum comparison of left carotid (LC) common artery before, during, and after EECP; The asterisk (*) is the primary peak (x1, y1) and secondary peak (x2, y2) of the power spectrum.

The power spectral density of the left CCA before, during, and after passive exercise is illustrated in Figure 2. These characteristics can reflect cardiac rhythm, systolic function, peripheral vascular impedance, and sympathetic activity (4, 15). Welch algorithm spectrum estimation is used to calculate the power spectral density in this study. Welch algorithm involves data segmentation and windowing and then averaging. First, the spectrum estimation of each segment is obtained, and then the total average is calculated (16). The raw blood flow velocity signal *x(n)* is divided into segments. The length of each segment of data is M, and it is allowed to overlap half of each segment of data. It is expressed as


(2) $$\begin{array}{l} L=\frac{N-M/2}{M/2}. \end{array}$$

The *i* segment is windowed and obtained by FFT. It is shown as


(3) $$\begin{array}{l} X_{i}\left(k\right)=\overset{M-1}{\underset{n=0}{\sum }}x_{i}\left(n\right)w\left(n\right)W_{M}^{kn}\text{,}k=0,1,...,N-1,\text{i=1}...\text{L,}\\ \;W_{M}=e^{-j\frac{2\pi }{M}}, \end{array}$$

where w is a tapering window, and *x*_*i*_(*n*) is the *i*th raw blood flow velocity signals.

The power spectrum of each segment of data was calculated as


(4) $$\begin{array}{l} p_{i}\left(k\right)=\frac{1}{MU}{\left|X_{i}\left(k\right)\right|}^{2},0\leq k\leq M-1\\ \;U=\frac{1}{M}\overset{M-1}{\underset{n=0}{\sum }}w^{2}\left(n\right)\\ w\left(n\right)=\frac{1}{2}\left(1-\cos\frac{2\pi n}{N-1}\right),0\leq n\leq N-1. \end{array}$$

The average power spectrum density was finally expressed as:


(5) $$\begin{array}{l} {\overset{-}{p}}_{i}\left(k\right)=\frac{1}{L}\overset{L}{\underset{i=1}{\sum }}p_{i}\left(k\right). \end{array}$$

Lee et al. proposed a new concept and calculation expression of energy ratio. The spectral energy in the 0-ihz range is defined as (17):


(6) $$\begin{array}{l} E_{i}=\int_{0}^{1}{\overset{-}{p}}_{i}\left(k\right)df. \end{array}$$

In this study, the energy ratio in different frequency bands was calculated in turn by the algorithm of dichotomy. According to the ultrasonic image, the acquisition frequency is 150 Hz, and the maximum frequency of energy spectrum is 75 Hz. Therefore, power spectral energy ratio (PSER) was defined as:


(7) $$\begin{array}{l} PSER\left(i\right)=\frac{E_{i}}{E_{75}}=\frac{\int_{0}^{1}{\overset{-}{p}}_{i}\left(k\right)df}{\int_{0}^{75}{\overset{-}{p}}_{i}\left(k\right)df}. \end{array}$$

It mainly shows the change of the fundamental frequency and harmonic components of the signal, and also the change of the energy ratio of each harmonic component to the total energy. Due to the energy of the signal mainly concentrated in the low-frequency band, PSER in the 0–2.05 Hz (p5) and PSER in the 0–0.87 Hz (p6) were extracted. Those characteristics have clinical significance for the kinetics of blood flow (3).

### Statistical Analysis

Results are shown as means ± SD. Normal distribution for all the frequency-domain characteristics was assessed by the Kolmogorov–Smirnov test. The independent *t*-test was conducted to compare differences in the basic characteristics of the two groups. Intragroup differences (before, during, and after 45 min-EECP values) were analyzed by repeated measures-ANOVA, and *post-hoc* analysis was used to determine differences between the three time periods. Between-groups differences of frequency-domain characteristics before, during, and after 45 min-EECP were analyzed by the two-factor ANOVA. SPSS version 20.0 (IBM SPSS Statistics, USA) was used for all statistical analyses, and *p* < 0.05 was considered as a measure of statistical significance.

## Results

The basic characteristics of the two groups are listed in Table 1. There were no significant differences in age, height, weight, BMI, and drinking (all *p* > 0.05), whereas, significant differences appeared in gender and risk factors between the two groups (all *p* < 0.05).

**Table 1:** Base information and major cardiovascular risk factors in two groups.

**Variables**	**CAD**	**Control**	***P*-value**
Number	21	21	
Age (year)	55.52 ± 7.53	53.95 ± 8.44	0.528
Male (percentage/*n*)	80.95 (17)	66.67 (7)	0.002
Height (cm)	165.55 ± 7.13	161.25 ± 9.47	0.113
Weight (kg)	69.59 ± 12.34	65.30 ± 13.31	0.292
BMI (kg/m^2^)	25.28 ± 3.47	24.36 ± 3.08	0.381
Hypertension (percentage/*n*)	85.71 (18)	0 (0)	0.000
Hyperlipidemia (percentage/*n*)	33.33 (7)	0 (0)	0.002
Hyperglycemia (percentage/*n*)	38.10 (8)	0 (0)	0.001
Smoking (percentage/*n*)	42.86 (9)	0 (0)	0.001
Drinking (percentage/*n*)	28.57 (18)	19.05 (4)	0.578

**Figure 3:**
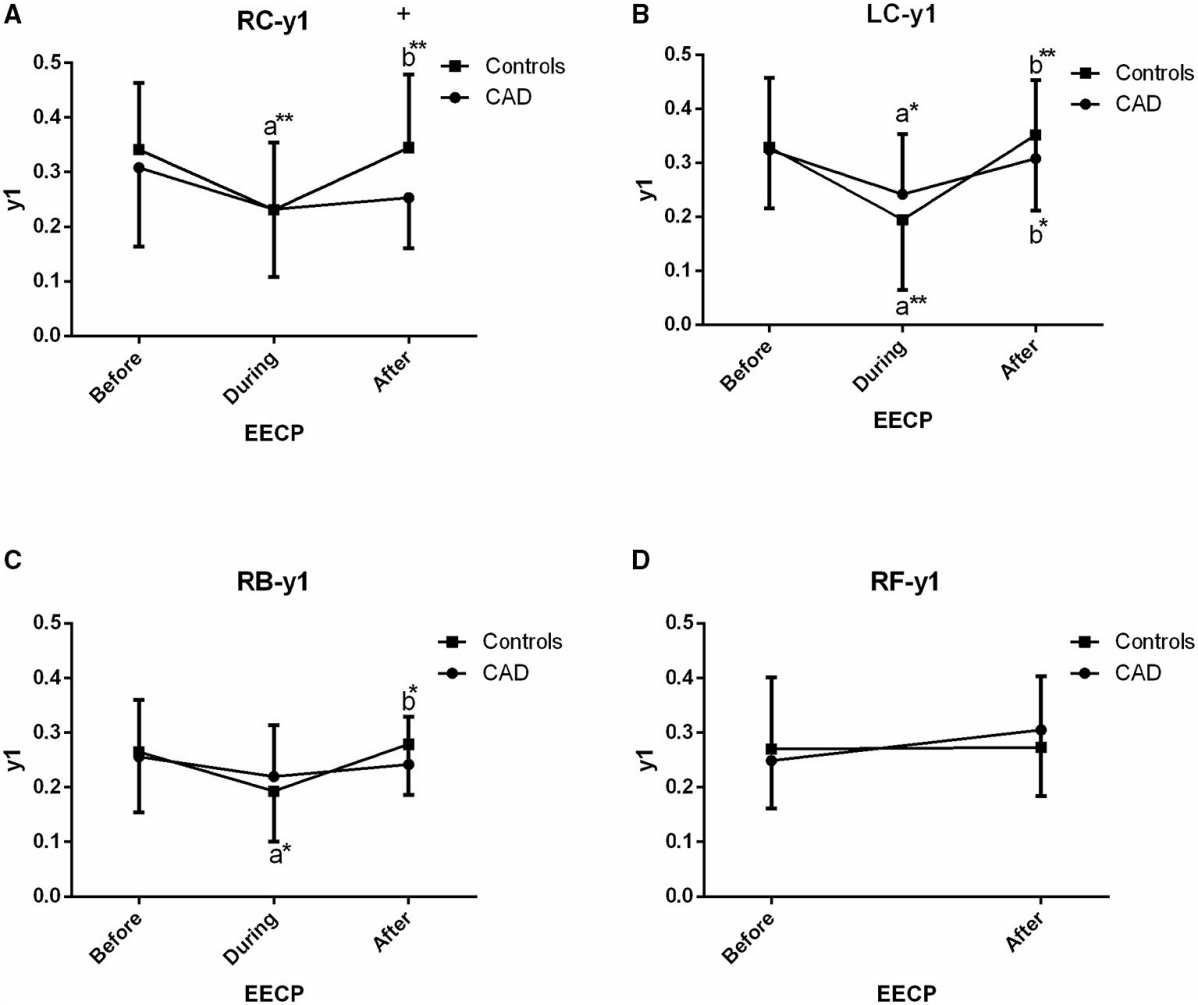
Effect of EECP on the first peak amplitude (y1) of LC and right carotid (RC) arteries **(A,B)**, right brachial (RB) artery **(C)** and right femoral (RF) artery **(D)** in patients with CAD and controls before, during and after 45-min EECP. a indicates significant differences in measurements obtained during EECP vs. pre-EECP and b indicates differences between post-EECP vs. during-EECP. “*” and “**” denote values of *p* < 0.5 and < 0.01, respectively.

**Figure 4:**
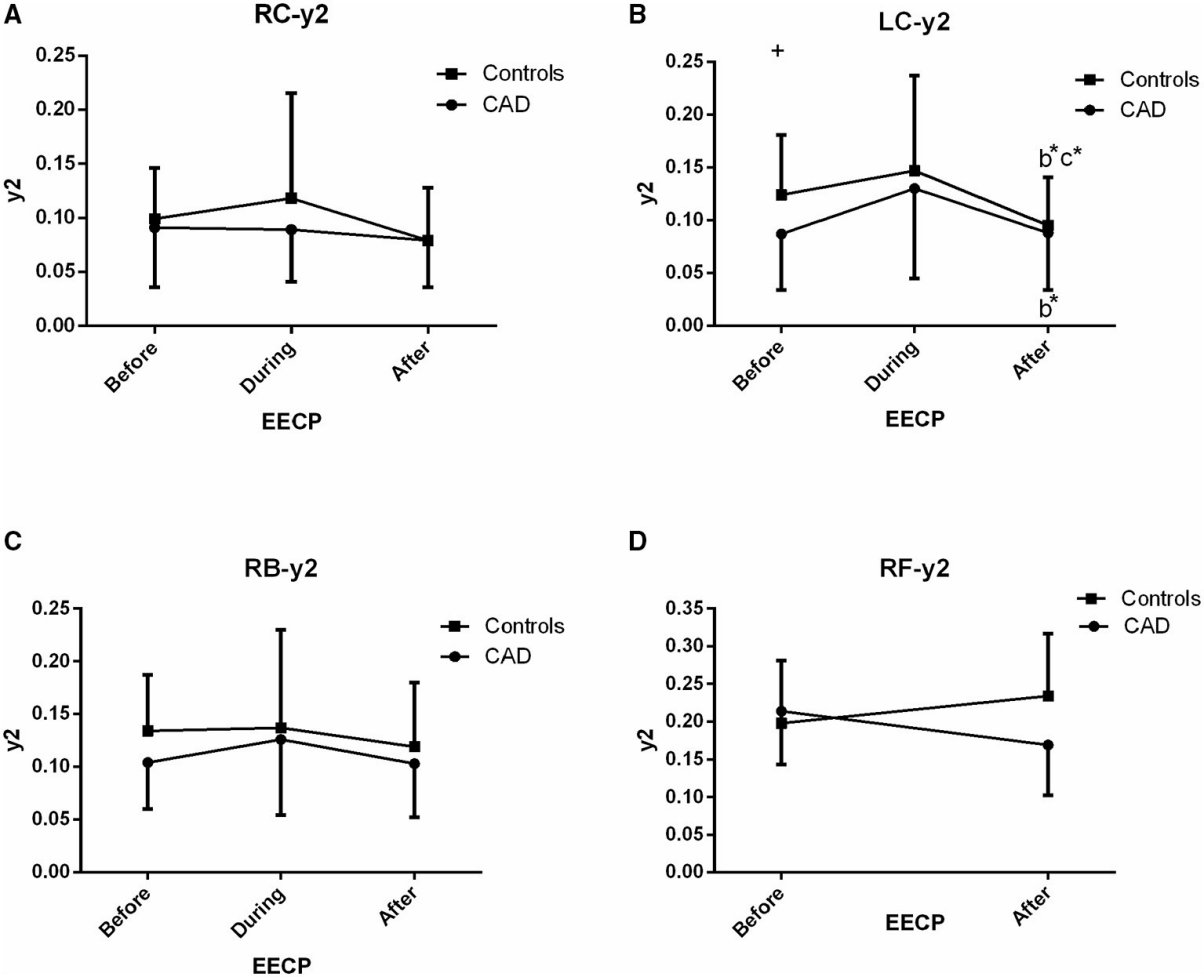
Effect of EECP on the second peak amplitude (y2) of the RC and LC arteries **(A,B)**, RB artery **(C)**, and RF artery **(D)** in patients with CAD and controls before, during and after 45-min EECP. b indicates differences between post-EECP vs. during-EECP and c refers to comparisons between post-EECP vs. pre-EECP. “*” denotes value of *p* < 0.5.

**Figure 5:**
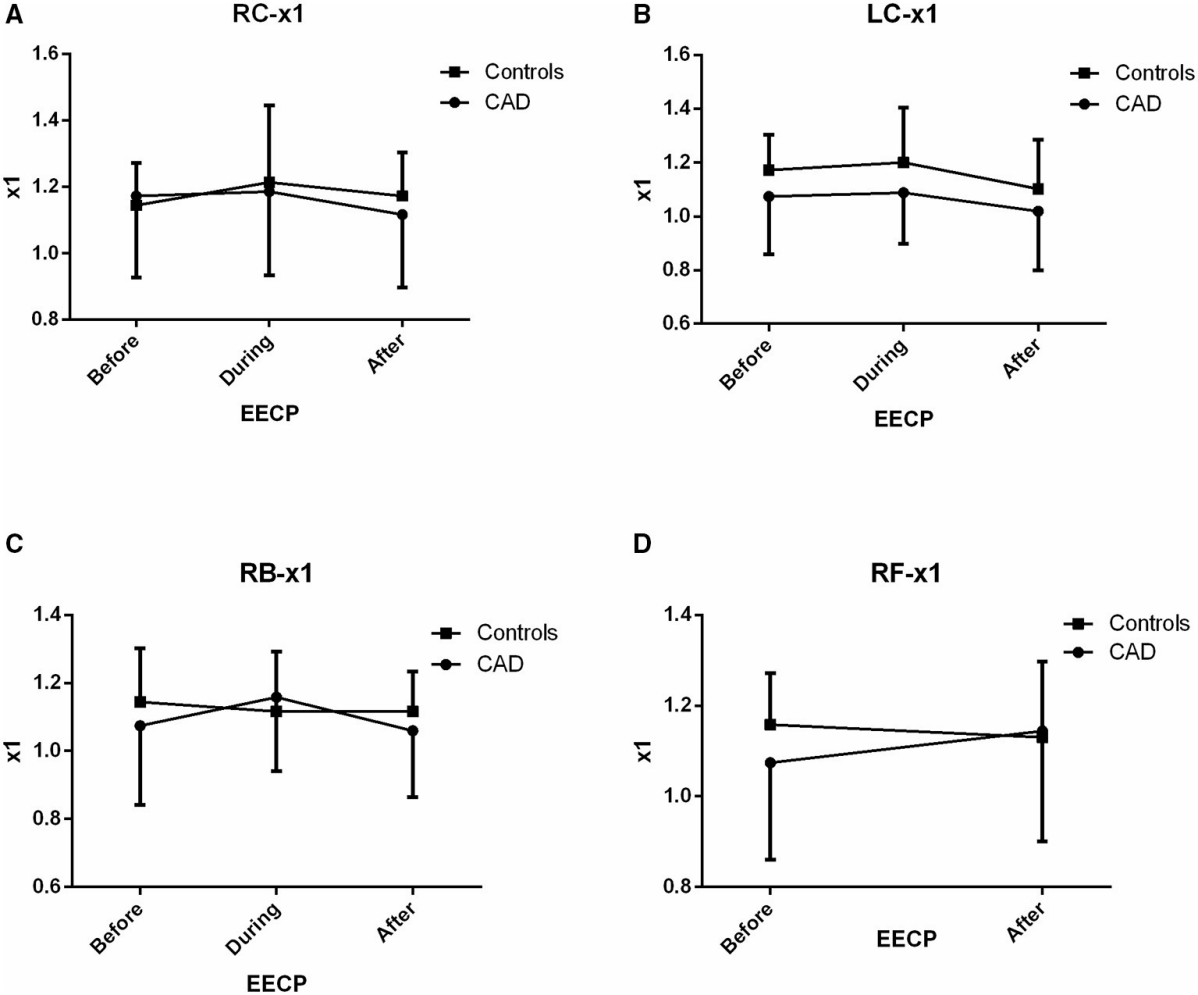
Effect of EECP on the fundamental frequency (x1) of LC and RC arteries **(A,B)**, RB artery **(C)**, and RF artery **(D)** in patients with CAD and controls before, during and after 45-min EECP.

**Figure 6:**
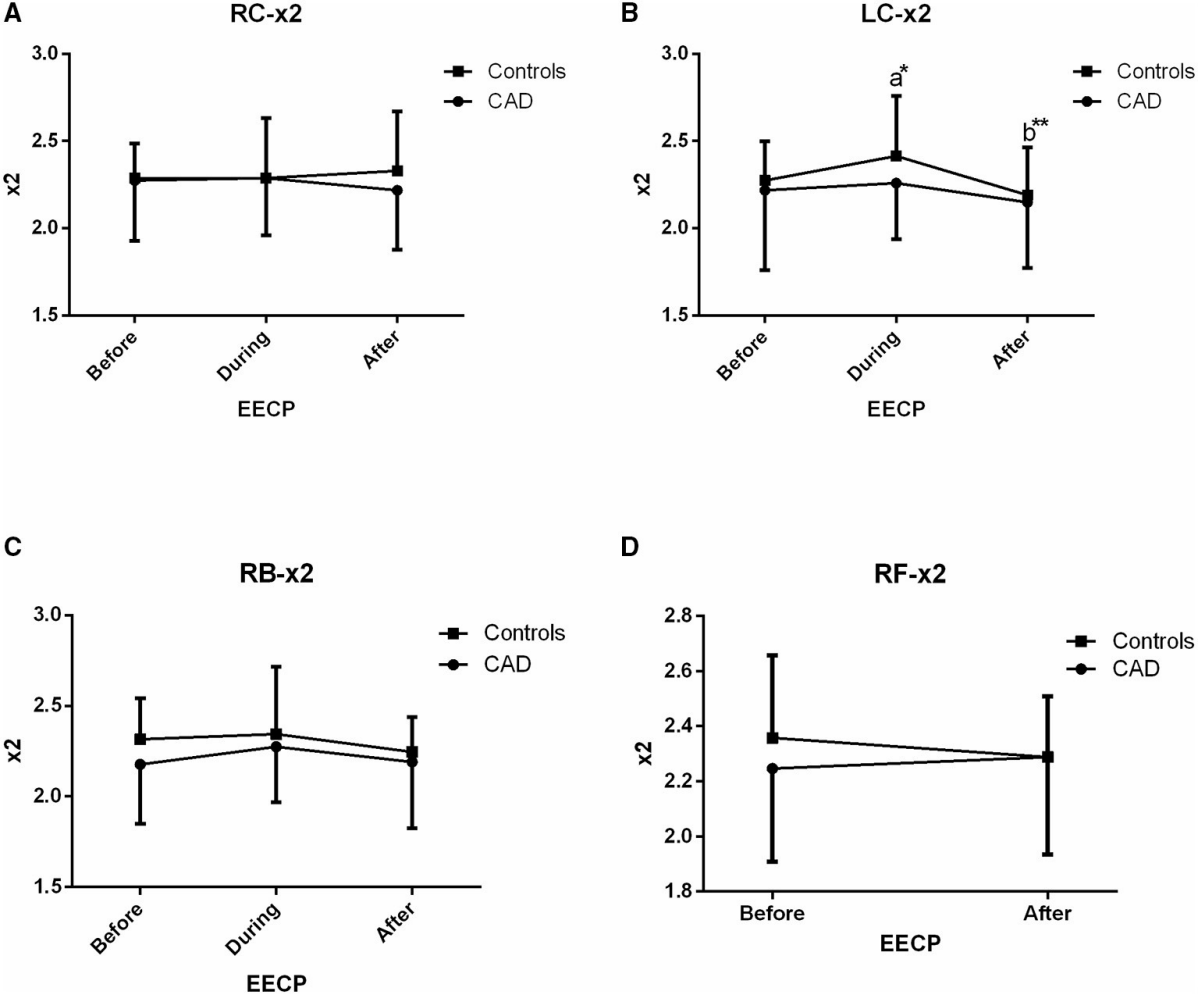
Effect of EECP on the second frequency (x2) of RC and LC artery **(A,B)**, RB artery **(C)**, and RB artery **(D)** in patients with CAD and controls before, during and after 45 min EECP. a indicates significant differences in measurements obtained during EECP vs. pre-EECP and b indicates differences between post-EECP vs. during-EECP. “*” and “**” denote values of *p* < 0.5 and < 0.01, respectively.

**Figure 7:**
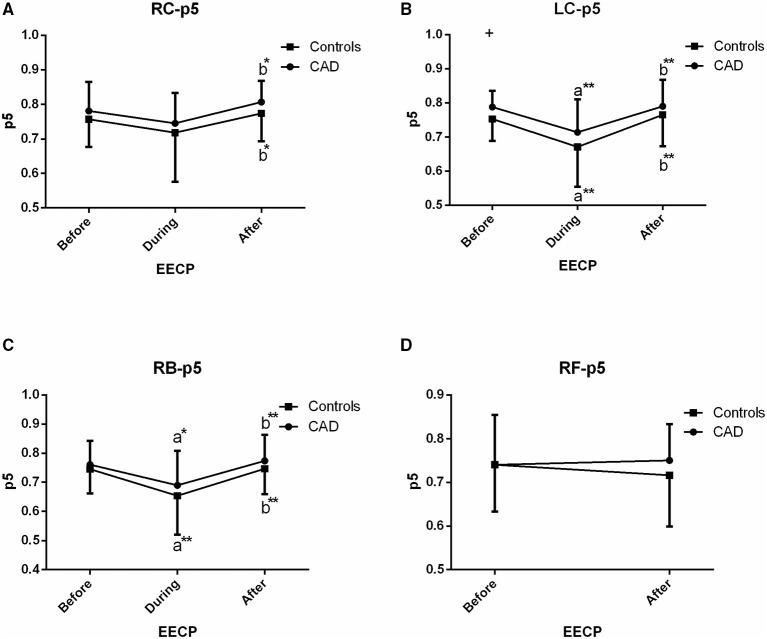
Effect of EECP on the PSER in the 0–2.05 Hz (p5) of LC and RC arteries **(A,B)**, RB artery **(C)**, and RF artery **(D)** in patients with CAD and controls before, during and after 45-min EECP. a indicates significant differences in measurements obtained during EECP vs. pre-EECP and b indicates differences between post-EECP vs. during-EECP. “*” and “**” denote values of *p* < 0.5 and < 0.01, respectively.

**Figure 8:**
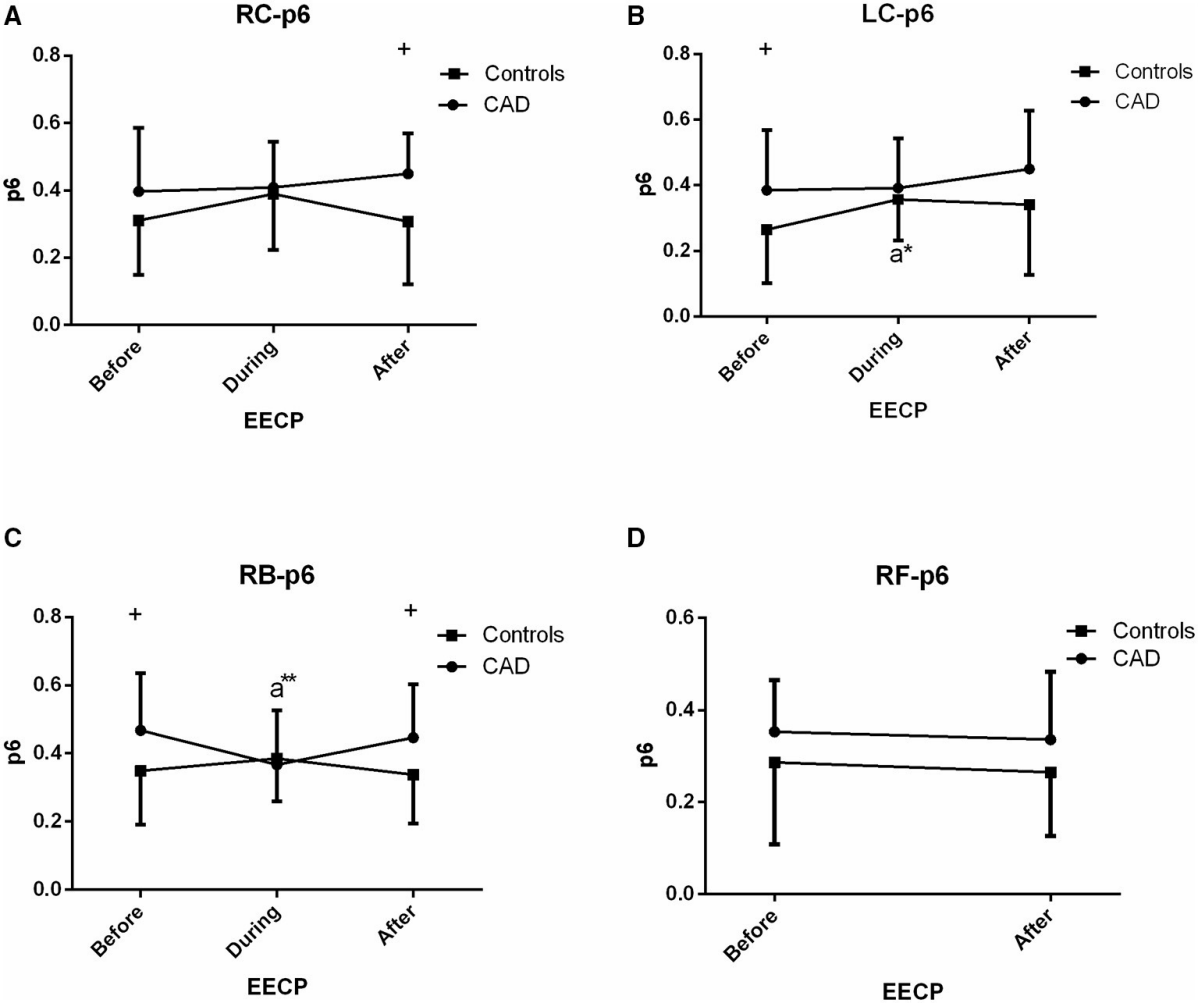
Effect of EECP on the PSER in the 0–0.87 Hz (p6) of LC and RC arteries **(A,B)**, RB artery **(C)**, and RF artery **(D)** in patients with CAD and controls before, during and after 45-min EECP. a indicates significant differences in measurements obtained during EECP vs. pre-EECP. “*” and “**” denote values of *p* < 0.5 and < 0.01, respectively.

The effect of EECP on the frequency-domain characteristics varied in each artery as did the differences between patients with CAD and controls. The results are illustrated in Figures 3–8, which compares the effects of EECP for each artery in controls and CAD patients separately and allows a direct comparison of CAD patients with controls.

### First Peak Amplitude (y1)

In the CCAs and RB, a reduction of y1 was seen during EECP, and it was significantly increased when the treatment ceased in controls (all *p* < 0.05, Figures 3B,C) whereas, the above responses of the LC just appeared (both *p* < 0.05, Figure 3B) in CAD patients. In addition, y1 of the RC in controls was significantly higher than that of CAD patients immediately after EECP (0.345 ± 0.134 vs. 0.253 ± 0.092, *p* = 0.013, Figure 3A).

### Second Peak Amplitude (y2)

There was a significant difference in y2 of the LC in the two groups immediately after EECP, while y2 was significantly lower only in the LC of controls than baseline (0.095 ± 0.046 vs. 0.124 ± 0.057, *p* = 0.014, Figure 4B). In addition, y2 in the LC of CAD patients was significantly lower than that of the controls at baseline (0.124 ± 0.057 vs. 0.087 ± 0.053, *p* = 0.037, Figure 4B).

### Fundamental Frequency (x1)

There was no significant difference in x1 of the two groups before, during, and immediately after EECP (all *p* > 0.05, Figure 5).

### Second Frequency (x2)

Only in the LC of controls, x2 was significantly increased and then markedly recovered at the end of EECP treatment (both *p* < 0.05, Figure 6).

### PSER in the 0–2.05 Hz (p5)

In the CCAs and RB, responses of p5 in CAD patients were similar to those of the controls, i.e., a fall during EECP and a sustained difference following EECP (all *p* < 0.05, Figure 7). However, no effect was seen in the RC in both the groups during EECP. Moreover, there was a significant difference in p5 of the LC between CAD patients and controls (0.753 ± 0.064 vs. 0.788 ± 0.048, *p* = 0.049, Figure 7).

### PSER in the 0–0.87 Hz (p6)

The p6 of the RB in CAD patients (0.367 ± 0.160 vs. 0.468 ± 0.168, *p* = 0.038, Figure 8) and the LC in controls (0.357 ± 0.125 vs. 0.265 ± 0.163, *p* = 0.015, Figure 8) was significantly reduced and increased during EECP compared with the baseline, respectively. Additionally, p6 of the LC and RB in CAD patients at baseline was significantly higher than those in the controls.

## Discussion

This study shows that in the LC, CAD patients and controls had similar responses in y1, y2, and p5 during passive exercise compared to the baselines. In addition, in the RC and RB, a significant response of p5 was also seen in the two groups. However, y1 only in the RC and RB and x2 only in the LC were significantly decreased in controls during passive exercise. Moreover, y2 of the LC was significantly lower in controls immediately after passive exercise than that at baseline. Furthermore, p6 was significantly reduced and elevated in the LC of controls and in the RB of CAD patients during passive exercise, respectively.

Frequency characteristic analysis, which contained a change in the fundamental frequency and harmonic components of the signal, can not only reflect the cardiovascular information but also predict cardiovascular diseases (1–3, 5). In this study, amplitude and frequency characteristics y1 and y2 in the LC of CAD patients were significantly decreased and increased during passive exercise, respectively. In addition, y1 in the RC of CAD patients was lower than those of controls after passive exercise.

However, among them, y1 only in the RC and RB and x2 only in the LC were significantly decreased in controls during passive exercise. Some studies had reported that frequency-domain characteristics showed the changes of impedance and flow waveforms (18–21). Meanwhile, McDonald found that the frequency of each harmonic is closely related to heart rate (HR), which is the fundamental frequency. Meanwhile, some studies also suggested the different phenomenon between central and peripheral pressure waves on the basis of wave reflection (22, 23).

In this study, y2 only in the LC had a significant difference in controls immediately after EECP than that at baseline. However, y2 in the LC of CAD patients was lower than those of controls after EECP. A study had found that the power of the 2nd harmonics was significantly increased. It might be associated with increased HR due to a reduction of vascular resistance after exercise (5). In addition, a study has also reported that changes of amplitude might happen as a consequence of reflection waveform, which related to the mismatched impedance and the ineffectively buffer the pulsatile component of flow in diabetes (2). Previous studies used Fourier analysis to evaluate blood flow velocity waveforms. It indicated that aging and hypertension led to an increase in the amplitude of Fourier frequencies <5 Hz (24, 25), with the largest differences of about 1 Hz, decreasing around 3 Hz, and elevating again from 4 to 5 Hz.

In our study, PSER in the 0–2.05 Hz and in the 0–0.87 Hz was investigated to reflect the change of the energy ratio of each harmonic component to the total energy. In the RC and RB, a significant effect of p5 was seen in CAD patients and controls. However, there was an opposite change in p6 between the two groups during EECP. Here, p6 in the LC and RB was significantly decreased and increased in controls and CAD patients during EECP, respectively. A study had reported that power frequency spectrum analysis will improve the early diagnosis and detection of early small flow disturbances which may be important in the clinical decision making for carotid artery disease (1, 26, 27). A study had also presented that pulse waveform analysis might be employed as a non-invasive measure of peripheral vascular responses, with evaluation to different cardiovascular diseases (28).

In the present study, we also found that p5 in the LC and p6 in the RB and LC of CAD patients were significantly higher than those of the controls at baseline. However, immediately after passive exercise, only p6 in the RC and RB of CAD patients was significantly higher than that of the controls. It was found that the ratio of energy distribution in different harmonic bands not only can be determined by the spectral harmonic energy ratio, but also quantify the spectral harmonic distribution of circulation information conveyed by the arterial pulse (23). In addition, a study showed that changes in the power spectrum of the pulse might be led to changes in blood flow to the vital arteries attached to the aorta (29, 30). Moreover, a study reported that pulse harmonics was associated with autonomic nervous modulation. However, a study showed that the sympathy–vagal balance can be assessed at rest and during recovery, but it is not supported during exercise (4). Increased power of harmonics of pulse wave might be caused by the decrease in vascular resistance (31). Therefore, responses of energy ratio may be affected by blood flow distribution, autonomic nervous modulation, and vascular resistance.

There were some limitations in the present study. Firstly, the sample size is relatively small; secondly, frequency-domain characteristics in the RF during EECP cannot be measured due to cuffs wrapped around the lower extremity; and thirdly, at the end of the treatment, the recovery time was minimal for the femoral and increased by 30 s−1 min for each subsequent measurement site.

## Conclusions

The present study showed that passive exercise reduces amplitude and frequency characteristics of carotid arteries, whereas, there was an opposite response of energy ratio characteristics in the LC and RB arteries to passive exercise between CAD patients and controls. In addition, energy ratio characteristics of spectrum in the brachial artery were markedly elevated in patients with CAD during passive exercise. Meanwhile, passive exercise only reduces amplitude characteristics of the left carotid artery in the control group. Responses of amplitude and frequency characteristics are sensitive in the healthy controls, whereas energy ratio characteristics are sensitive in patients with CAD after passive exercise intervention.

## Data Availability Statement

The raw data supporting the conclusions of this article will be made available by the authors, without undue reservation.

## Ethics Statement

The studies involving human participants were reviewed and approved by Medical Ethics Committee of the Eighth Affiliated Hospital of Sun Yat-sen University. The patients/participants provided their written informed consent to participate in this study.

## Author Contributions

YZ proposed the scientific problems and contributed to the revision and final version of the manuscript. YZ and XZ designed and collected the experimental data and processed and calculated the data. XZ conducted the statistical analysis and wrote the draft manuscript. All authors contributed to the article and approved the submitted version.

## Conflict of Interest

The authors declare that the research was conducted in the absence of any commercial or financial relationships that could be construed as a potential conflict of interest.

## Publisher's Note

All claims expressed in this article are solely those of the authors and do not necessarily represent those of their affiliated organizations, or those of the publisher, the editors and the reviewers. Any product that may be evaluated in this article, or claim that may be made by its manufacturer, is not guaranteed or endorsed by the publisher.
